# Diurnal Cortisol in Left-Behind Adolescents: Relations to Negative Family Expressiveness and Internalizing Problems

**DOI:** 10.3389/fpubh.2022.844014

**Published:** 2022-05-10

**Authors:** Man Li, Qili Lan, Lirong Qiu, Yidan Yuan, Fengjiao He, Chen Zhang, Linlin Zhang

**Affiliations:** ^1^Key Research Base of Humanities and Social Sciences of the Ministry of Education, Tianjin Normal University, Academy of Psychology and Behavior, Tianjin, China; ^2^Faculty of Psychology, Tianjin Normal University, Tianjin, China; ^3^Tianjin Social Science Laboratory of Students' Mental Development and Learning, Tianjin, China; ^4^School of Psychological and Cognitive Science, Beijing Key Laboratory of Behavior and Mental Health, Peking University, Beijing, China; ^5^Key Laboratory of Learning and Cognition, School of Psychology, Capital Normal University, Beijing, China

**Keywords:** HPA axis, diurnal cortisol, left-behind adolescents, family expressiveness, internalizing problems

## Abstract

Despite the accumulating evidence for increased risks for behavioral problems in left-behind adolescents in China, little research has explored their HPA axis functioning, which is hypothesized to play a central role in the association between early adversity and health. In the present study, we designed a longitudinal study to examine HPA axis function in left-behind adolescents and its mediating role in the association between family emotional expressiveness and internalizing problems. Participants were 81 adolescents (44 female; 37 male) aged 11–16 years. Salivary cortisol samples were collected six times a day for two consecutive days on regular school days. Negative family expressiveness (NFE) and internalizing problems were measured using self-report questionnaires. The results showed that NFE was negatively associated with diurnal cortisol, and diurnal cortisol was negatively associated with internalizing problems. Further analysis showed that diurnal cortisol secretion measured by AUC (area under the curve) mediated the association between NFE and internalizing problems. Our findings extended the existing literature about left-behind children via a psychoneuroendocrinological perspective, documenting the negative consequences of the family environment for youth health and development.

## Introduction

In China, more than 61 million children and adolescents aged 16 or younger are left behind in rural areas due to their parents' engagement in rural-to-urban labor migration (1, 2). Adolescence is a period of rapid physical, psychological, and social development. Previous studies found that deteriorated parent–child relations induced by parents' migration led to risks for left-behind adolescence (3–7). Specifically, left-behind adolescence reported higher levels of internalizing problems, such as anxiety, depression, and social withdrawal (8, 9). Internalizing problems in children have been associated with serious long-term consequences during the adult years, such as major depression (10–12), substance abuse (11), anxiety (12, 13), suicidal ideation (13), and antisocial behaviors (10). Therefore, identifying mechanisms that lead to internalizing problems among left-behind adolescents is essential for designing effective intervention or prevention programs.

## Family Emotional Expressiveness and Internalizing Problems

Ecological systems theory holds that human development is a process of self-centered interaction with the surrounding environmental system (14). The family, as a central part of the microsystem, plays an important role in adolescents' development (15). Family emotional expressiveness refers to the style of verbal or non-verbal expression of emotions in a family and is an important mechanism through which caregivers' emotional styles affect children's emotional regulation ability (16). Studies of normative families have shown that children living in families with high levels of negative emotional expressiveness are more likely to have poor social adaptation (17), inadequate emotional regulation (18), and more problem behaviors (19). In left-behind children, parental absence as a result of parents' migration weakens parent–child bonding, reduces parent-adolescent communication, and thereby likely induces more negative emotional expressiveness in parent–child interactions (20–22). However, family emotional expressiveness has rarely been examined in left-behind adolescents, overlooking the fact that left-behind adolescents maintain communication with their parents through various channels (i.e., telephone calls and messages).

## The Role of HPA Axis Function

The hypothalamic–pituitary–adrenal (HPA) axis is hypothesized to play a central role in the association between early adversity and health (23–26). Across the day, cortisol levels follow a circadian rhythm as cortisol is released in pulses. Cortisol levels are generally higher in the morning, with peak levels occurring ~30 min after waking, followed by a decline throughout the day until reaching a nadir in the evening with sleep (25). There are several indicators that reflect the function of the HPA axis, such as cortisol awakening response (CAR), diurnal slope, and total cortisol output (AUC). The CAR influenced by sleep may represent adrenal sensitivity to ACTH (27, 28). The diurnal slope reflects the circadian rhythm of cortisol, which is associated with the suprachiasmatic nucleus (SCN) of the hypothalamus (29). The AUC approximates total cortisol production throughout the day, which aggregates indices of circadian functioning with responses to stress across the day (30).

A harsh family environment due, for example, to poverty tends to expose children to higher levels of stress and leads to abnormal HPA axis activity (31). For example, some researchers found that children from low-income families had higher cortisol levels (32, 33). In contrast, some studies found that adverse family factors (such as emotional maltreatment) were associated with lower cortisol levels (34–36). The contradictory results of the studies on the relationship between adverse family environments and cortisol levels conform to two opposing theories: the hypercortisolism hypothesis and the hypocortisolism hypothesis. The hypercortisolism hypothesis suggests that chronic stress causes high HPA axis sensitivity and increases cortisol levels (37), while the hypocortisolism hypothesis suggests that HPA axis activation decreases under chronic stress, with lower cortisol levels and a flatter diurnal rhythm (38). Previous studies have found that adolescents who lived with higher reported parent–child negativity showed higher bedtime cortisol (39). However, little is known about the relationship between the family environment in left-behind adolescents and HPA axis functioning.

An increasing number of studies have suggested that altered HPA axis activity may be a precursor to increased internalizing problems (40). In young children, higher total cortisol output and a flatter diurnal rhythm have been found to be related to elevated internalizing problem symptoms at 36 months (41). Likewise, a meta-analytic review showed that depressed children and adolescents showed increased morning cortisol (42). Adolescents with high basal cortisol were found to exhibit increasingly severe internalizing problems over a 2-year period (43). Despite the above studies showed the association between high cortisol levels and problem behaviors, it should be acknowledged that higher levels of cortisol are not always detrimental and can be adaptive in certain situations. For example, studies have found that high levels of cortisol induced by exercise are beneficial to mental health (44).

In some studies, cortisol played a mediating role in the relationships among chronic stress, poor parenting behavior and problem behaviors (45, 46). A previous study indicated that children's cortisol levels were one of the mechanisms by which parenting factors influenced internalizing problems (47). Taken together, it is reasonable to speculate that cortisol may also be an important physiological mechanism linking a harsh family environment and internalizing problems in left-behind adolescents.

## The Present Study

To date, there are few studies investigating left-behind adolescents' HPA axis functioning and its role in the relation between family adversity and mental health. The current study was designed as a longitudinal study to fill several gaps in the literature. The first goal was to investigate the association between negative family expressiveness and various indicators of HPA axis functioning in left-behind adolescents. By collecting saliva from the subjects six times a day across two consecutive days, we were able to calculate the AUC, diurnal slope, and CAR to gain a comprehensive understanding of left-behind adolescents' HPA axis functioning. Second, we also examined the mediating role of the HPA axis functioning between negative family expressiveness and internalizing problems. Based upon the limited existing literature on healthy youth (34–36), we hypothesized that higher negative family expressiveness would be associated with indicators of a downregulated HPA axis: lower cortisol awakening response (CAR), lower total daily cortisol output (AUC) and flatter diurnal slope. Furthermore, we hypothesized that abnormal diurnal cortisol levels would mediate the relationship between family expressiveness and internalizing problems in left-behind adolescents.

## Methods

### Participants

Participants were recruited from a middle school in rural Northwest China. The sample included 81 adolescents (female *n* = 44; male *n* = 37) between 11 and 16 years of age (*M* = 13.99; *SD* = 0.90 years). Their parents were between 32 and 40 years old. At least one of the parents of each child had been away from home for more than 6 months. The duration of parental absence ranged from 0 to 15 years (*M*_Father_ = 6.22, *M*_Mother_ = 5.16 years). The proportion of participants with both parents out, only father out, only mother out were 75.9, 15.2, and 8.9%, respectively. In terms of the highest education level achieved, 21% of the fathers did not go to primary school, 6.2% attended primary school for only a brief period, 37% finished primary school, 43.2% junior high school, 6.2% high school, and 1.2% college. Regarding the mothers, 35.8% did not go to primary school, 4.9% attended primary school for only a brief period, 33.3% of the mothers completed primary school, 22.2% junior high school, and 1.2% college.

### Procedures

The study was approved by the ethical committee of Tianjin Normal University. All subjects and one of their parents signed the informed consent form. Data were collected in two phases. Family emotional expressiveness and problem behavior data were collected at the participants' home during Chinese New Year (T1, January 2017). Six months later (T2), problem behaviors were reassessed collectively at school, and the subjects' saliva samples were collected six times a day for two consecutive days on regular school days. The questionnaires were completed on computers or smartphones.

### Measures

#### Demographic Questionnaire

The students reported their demographic information, including their gender, grade, the total number of years their parents were away from home and their parents' highest level of education.

#### Negative Family Expressiveness

The negative expressiveness subscales of the Family Expressiveness Questionnaire (FEQ) was used (48). The subscale includes 20 items which describe an individual's history of negative family expression. It contains two dimensions: dominance and submissiveness. The negative dominance dimension captures expressions of criticism, contempt and anger in the family, whereas the negative submissiveness dimension captures expressions of sorrow, embarrassment, and disappointment in the family. Both dimensions assess negative emotional expressiveness in the family and were aggregated to form a composite score of negative family expressiveness. Individuals circled a rating for each item on a nine-point scale ranging from (1) “*not at all frequently in my family”* to (9) “*very often frequently in my family”* to indicate the frequency with which they experienced the expressive situation while they were growing up in comparison to peers in other families. The internal consistency coefficient of the subscale was between 0.81 and 0.92 in previous studies of Chinese children (49). In the present study, the internal consistency coefficient was 0.82.

#### Internalizing Problem Behaviors

Adolescents' internalizing problem behaviors were measured using the Youth Self-Report Form [YSR; (50)]. Subjects were asked to rate a list of items describing behavioral characteristics based on their situation in the past 6 months. A 3-point Likert scale was used (0 = “*not true”*, 1 = “*somewhat or sometimes true”*, 2 = “*very true or often true”*). The behaviors evaluated include withdrawal, anxiety, depression and social problems. Item scores were averaged to form a composite of internalizing problem behavior, with higher scores reflecting more internalizing problems. In our study, the internal consistency coefficients of internalizing problem behavior were 0.75 (T1) and 0.83 (T2).

#### Diurnal Cortisol

Salivary cortisol levels were measured as a marker of HPA axis activity. Saliva was collected using a cotton swab, and the participants were instructed to place between their tongue and cheek for ~2 min until the swab was completely saturated. The saliva swab was collected in a plastic tube, which was placed directly on ice and stored at −20°C. Saliva samples were assayed in duplicate following standard radioimmunoassay procedures with no modifications (Salivary Cortisol ELISA Kit, Salimetrics, USA) at the Physiological Psychology Experiment Platform of the Institute of Psychology, CAS. The test uses 25 μl of saliva per determination, has a lower limit of sensitivity of 0.007 μg/dl and standard curve range from 0.012 to 3.0 μg/dl. The intra-assay coefficients of variation for the kit ranged from 3.0 to 10%. Saliva samples were taken at ~6:00 a.m., 6:30 a.m., 9:30 a.m., 11:40 a.m., and 3:50 p.m. and 10:00 p.m. on two consecutive days. For the first two time points, saliva was collected immediately after the subject woke up and half an hour later at home; the specific time was recorded by the subject, and the subject then brought the saliva to the school and gave them to the research assistants. In order to prevent students from forgetting to collect saliva or to mark the time of saliva collection at home, we reminded students the day before they collected saliva. In addition, we also reminded their caregivers to assist in saliva collection and labeling. The children's reported first timestamps of saliva collection ranged from 5:50 to 6:10 a.m. The remaining four saliva samples were collected by the children at school.

### Missing Data

Due to the time interval between measurements for all the variables in the study, there were some missing data. Family expressiveness and problem behavior data were not available at T1 for ten children due to incomplete questionnaires. There were two children who had no problem behavior data at T1 or T2. Due to insufficient salivary volume, there was one missing cortisol value separately at each time of the day. To make effective use of the data and reduce biases, the missing values of all the variables involved were treated using the full information maximum likelihood (FIML) method in this study.

### Data Analysis

Data were analyzed in SPSS 26.0 and M*plus* 8.30. Cortisol at each time point was averaged across the 2 days to increase the reliability of the measurements (51). The CAR was calculated by subtracting the mean waking cortisol concentration from the mean 30-min postwaking cortisol concentration. The area under the curve with respect to ground (AUC) was the summation of the five trapezoids consisting of the six cortisol measurements and the time distance between the measurements. It was calculated following methods set forth by Pruessner et al. (52). The diurnal slope was calculated by computing the difference between cortisol at bedtime and cortisol at waking divided by the cortisol at waking. A repeated ANCOVA was used to test the diurnal cortisol pattern at all of the cortisol time points. We used hierarchical linear regression to test the continuous relation between NFE and AUC, as well as with the CAR and diurnal cortisol slope. Then, we performed a mediation analysis to test the mediation effect of diurnal cortisol between NFE and internalizing problems. The indirect effects were evaluated using bias-corrected bootstrap confidence intervals with 5,000 draws. All cortisol data were log transformed in the hierarchical linear regression and mediation analysis.

## Results

### Preliminary Analyses

**Figure 1:**
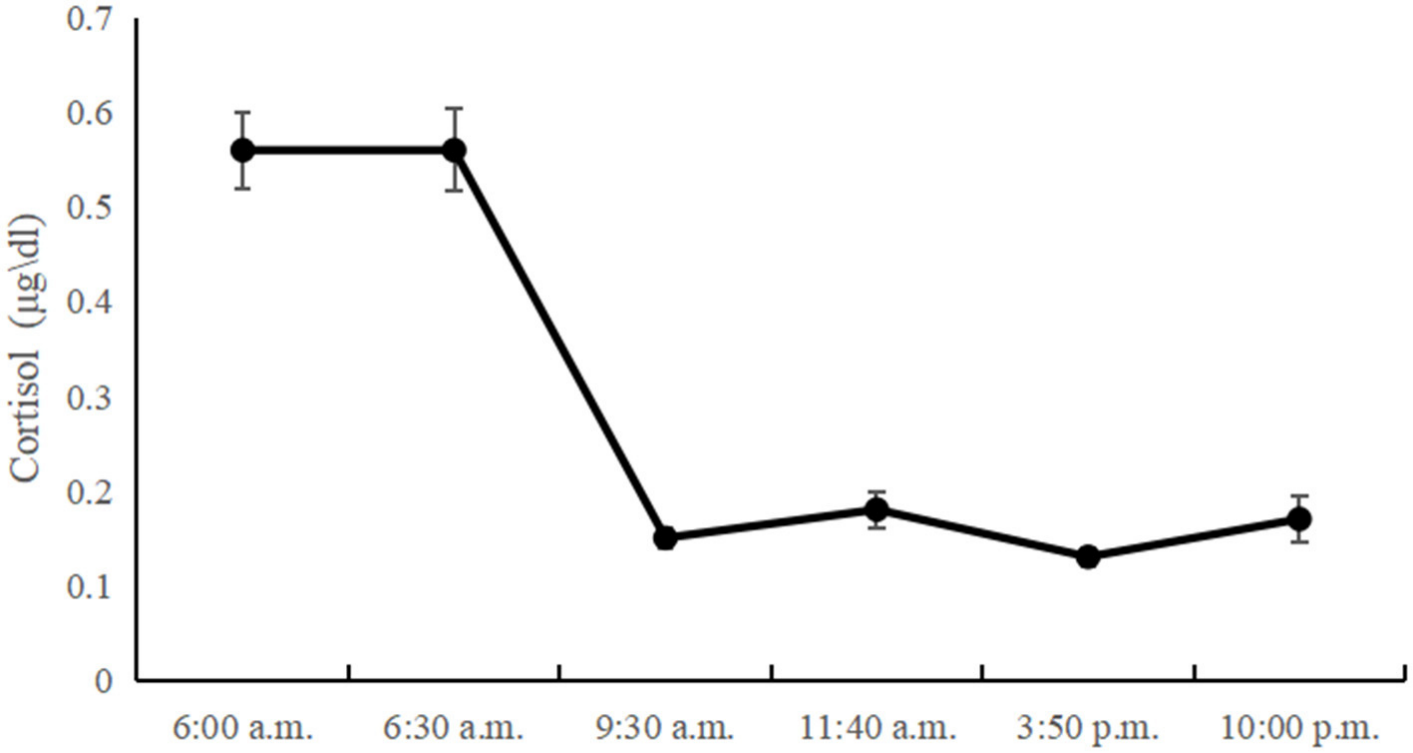
The diurnal cortisol levels at all time-points in left-behind adolescents.

Table 1 presents the descriptive statistics and bivariate correlations among the study variables. The diurnal cortisol levels showed a significant time effect [*F*_(3, 345)_ = 3.86; *p* < 0.05]. Morning cortisol levels peaked and gradually declined until the last time point. However, there was no significant CAR in left-behind adolescents, and the diurnal cortisol levels showed a sharp decline between 6:30 a.m. and 9:30 a.m., with little change between 9:30 a.m. and 10:00 p.m. The diurnal cortisol levels at all time-points are showed in Figure 1.

**Table 1:** Correlations between key variables.

	**Mean**	**SD**	**1**	**2**	**3**	**4**	**5**	**6**	**7**	**8**	**9**	**10**	**11**	**12**	**13**	**14**	**15**
Gender																	
Age	13.99	0.90	0.07														
YM	4.78	3.89	0.26^*^	0.04													
YF	5.92	4.79	0.10	0.30^**^	0.45^**^												
T1-NFE	3.90	1.27	0.34^**^	0.09	0.06	0.02											
T1 cortisol	0.56	0.36	−0.01	−0.24^*^	0.17	0.00	−0.27^*^										
T2 cortisol	0.56	0.39	0.02	−0.16	0.03	−0.04	−0.16	0.69^**^									
T3 cortisol	0.15	0.09	−0.07	−0.15	−0.02	−0.001	−0.20	0.54^**^	0.64^**^								
T4 cortisol	0.18	0.17	0.13	−0.25^*^	0.10	−0.11	−0.24^*^	0.44^**^	0.40^**^	0.38^**^							
T5 cortisol	0.13	0.09	0.22^*^	−0.13	−0.03	−0.13	−0.16	0.49^**^	0.53^**^	0.48^**^	0.38^**^						
T6 cortisol	0.17	0.23	0.16	−0.09	0.09	0.04	0.02	0.26^*^	0.46^**^	0.47^**^	0.40^**^	0.29^*^					
AUC	199.56	124.47	0.13	−0.22	0.05	−0.06	−0.19	0.67^**^	0.85^**^	0.73^**^	0.67^**^	0.69^**^	0.29^*^				
Diurnal slope	0.64	0.40	−0.14	−0.03	0.09	0.12	−0.15	0.19	−0.12	−0.24^*^	−0.16	−0.18	0.06	−0.40^**^			
CAR	0.004	0.30	0.04	0.08	−0.16	−0.04	0.11	−0.29^**^	0.48^**^	0.20	−0.01	0.11	−0.18	0.31^**^	−0.40^**^		
T1- internalizing problems	1.47	0.35	0.25^*^	−0.05	0.03	0.04	0.45^**^	−0.20	−0.13	−0.28^*^	−0.18	−0.17	0.11	−0.21	0.04	0.08	
T2- internalizing problems	1.47	0.40	0.19	0.03	0.07	0.08	0.28^*^	−0.32^**^	−0.24^*^	−0.31^**^	−0.24^*^	−0.27^*^	−0.17	−0.28^*^	−0.05	0.08	0.44^**^

*YM, Years of mother's absence; YF, Years of father's absence; NFE, Negative family expressiveness; T1, 6:00 a.m.; T2, 6:30 a.m.; T3, 9:30 a.m.; T4, 11:40 a.m.; T5, 3:50 p.m.; T6, 10:00 p.m.; AUC, Area under the curve; CAR, cortisol awakening response*;

* *p < 0.05*;

** *p < 0.01*.

**Figure 2:**
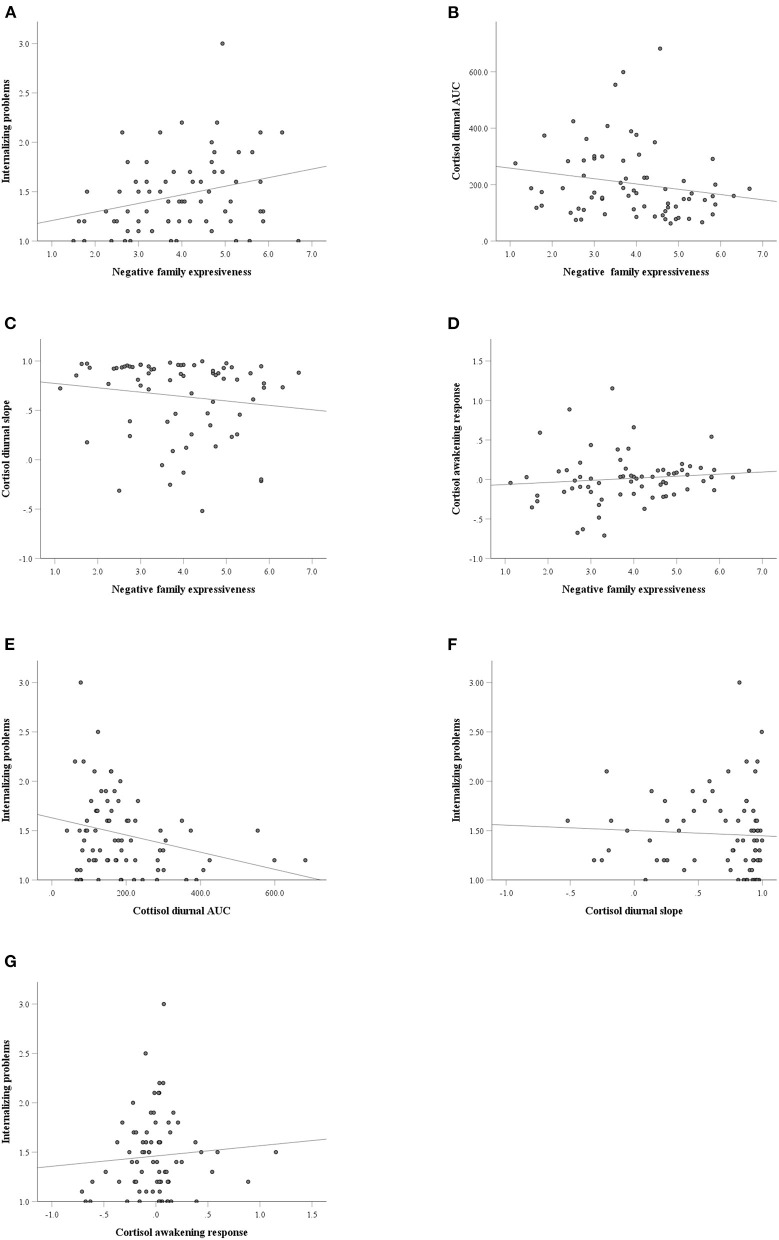
The scatterplots of the correlations between the pairs of variables. **(A)** Negative family expressiveness and internalizing problems; **(B)** Negative family expressiveness and cortisol diurnal AUC (area under the curve); **(C)** Negative family expressiveness and cortisol diurnal slope; **(D)** Negative family expressiveness and cortisol awakening response; **(E)** Cortisol diurnal AUC and internalizing problems; **(F)** Cortisol diurnal slope and internalizing problems; **(G)** Cortisol awakening response and internalizing problems.

Negative family expressiveness was associated with lower morning and noon cortisol levels (*p* < 0.04) and higher levels of internalizing problems at T1 (*p* < 0.01) and T2 (*p* < 0.02). The cortisol level at 9:30 a.m. was negatively associated with internalizing problems at T1 (*p* < 0.05). The cortisol levels at 6:00 a.m., 6:30 a.m., 9:30 a.m., 11:40 a.m., 3:50 p.m. and AUC were negatively associated with internalizing problems at T2. The scatterplots of the correlations between the pairs of variables are shown in Figure 2.

### Regression Analysis of Negative Family Expressiveness on Diurnal Cortisol in Left-Behind Adolescents

To investigate the effects of NFE on diurnal cortisol, we conducted a regression analysis with age, gender, years of mother's absence and years of father's absence controlled. For AUC, the results showed that NFE significantly predicted AUC [*b* = −0.051, SE = 0.022, 95% CI (−0.094, −0.007)]. However, the results showed that NFE did not predict the diurnal slope [*b* = −0.003, SE = 0.031, 95% CI (−0.059, 0.066)] or CAR [*b* = 0.023, SE = 0.028, 95% CI (−0.033, 0.077)]. The results were illustrated in Table 2.

**Table 2:** Linear regression for negative family expressiveness with AUC, diurnal slope and CAR.

	**Predictors**	***B***	***SE***	**95% CI**
AUC	Gender	0.093	0.060	−0.027, 0.210
	Age	−0.043	0.333	−0.111, 0.019
	YM	−0.005	0.008	−0.021, 0.010
	YF	−0.002	0.006	−0.014, 0.009
	NFE	−0.051	0.022	**−0.094**, **−0.007**
Diurnal slope	Gender	0.846	0.306	0.244, 1.450
	Age	0.102	0.199	−0.241, 0.541
	YM	−0.009	0.040	−0.090, 0.065
	YF	−0.301	0.379	−1.067,0.432
	NFE	−0.003	0.031	−0.059, 0.066
CAR	Gender	0.027	0.080	−0.123, 0.189
	Age	0.025	0.044	−0.066, 0.110
	YM	−0.014	0.009	−0.030, 0.005
	YF	0.001	0.008	−0.017, 0.016
	NFE	0.023	0.028	−0.033, 0.077

*AUC, Area under the curve; YM, Years of mother's absence; YF, Years of father's absence; NFE, Negative family expressiveness; CAR, cortisol awakening response. The bold values means NFE is a significant predictor of AUC*.

### Family Expressiveness and Internalizing Problems in Left-Behind Adolescents: The Mediating Role of Diurnal Cortisol

**Figure 3:**
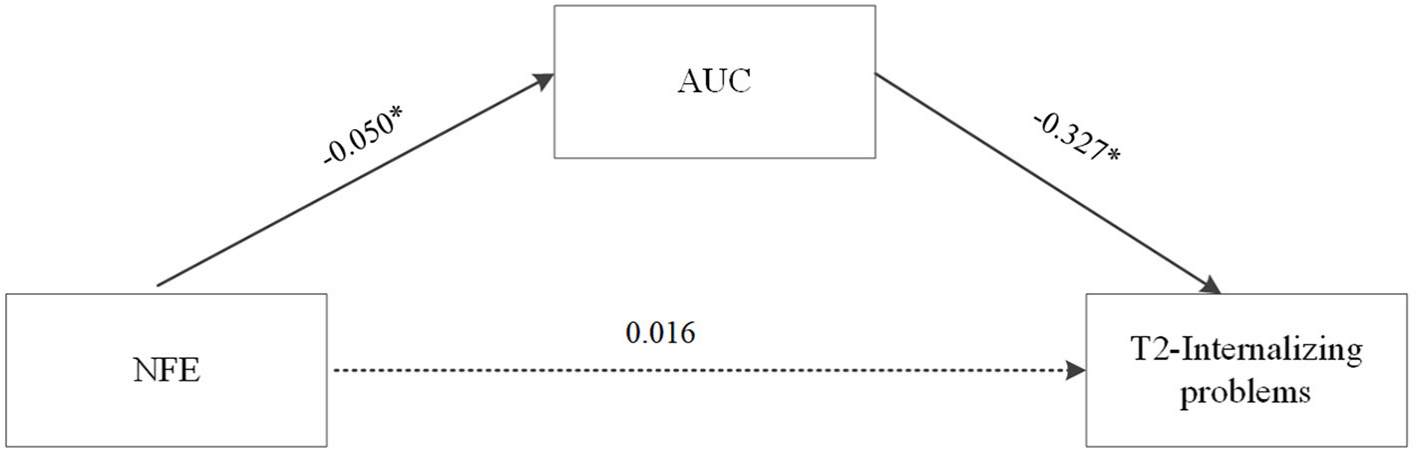
The mediating role of AUC in the relationship between NFE and internalizing problem behaviors at T2 in left-behind adolescents. Unstandardized path coefficients were shown (NFE, negative family expressiveness, *p < 0.05).

Based on the correlation and regression analyses, the mediating role of AUC in the relationship between NFE and problem behaviors at T2 in left-behind adolescents was further tested with age, gender, years of mother and father absence, internalizing problems at T1 controlled. Using 5,000 bootstrap samples selected from the random sampling test, the results showed that the direct effect of NFE on internalizing problems at T2 was not significant [*b* = 0.016, *SE* = 0.044, 95% CI = (−0.077, 0.097)]. The indirect effect of NFE on internalizing problems at T2 was significant [*b* = 0.016, *SE* = 0.013, 95% CI = (0.000, 0.054)], indicating that the AUC had a mediating effect on the relation between NFE and internalizing problems at T2. The mediation model is shown in Figure 3.

## Discussion

The purpose of this study was to identify the association between negative family expressiveness and various indicators of HPA axis functioning, including CAR, diurnal slope, and AUC in left-behind adolescents. Furthermore, we designed a longitudinal study to test the mediating effect of HPA axis functioning between the NFE and left-behind adolescents' internalizing problems. The results showed that NFE was negatively correlated with diurnal cortisol levels. Further analysis showed that high NFE predicted a lower diurnal cortisol level in left-behind adolescents. Furthermore, the NFE score significantly predicted internalizing problems at T2, with the AUC acting as a mediator. Our study provided evidence for the effects of NFE on internalizing problems from a psychoneuroendocrinological perspective and enriched the existing research on left-behind adolescents.

### Left-Behind Adolescents' HPA Axis Functioning

Previous studies have indicated that cortisol is high in the morning, declines throughout the day, and reaches a nadir in the evening with sleep (53). Our results show that left-behind adolescents' diurnal cortisol generally follows the same pattern. However, the diurnal cortisol pattern of left-behind adolescents is somewhat different from that of other adolescents. Previous research found that there was no significant difference between morning cortisol levels and cortisol levels 3 h later but reported a relatively steep drop after 8 h, followed by a more moderate decrease until the lowest point at bedtime (54). In other words, the decrease in cortisol concentration typically occurred in the middle of the day. Our study found that the rapid decrease in cortisol levels occurred in the morning, with little change between 9:30 a.m. and 10:00 p.m. The reason for the inconsistencies across studies may be the different developmental periods of the children: prior studies' children were in late adolescence, and ours were in mid-adolescence. In addition, we speculated that the daily variation pattern of cortisol presented in this study might be unique to left-behind adolescents. To the best of our knowledge, there are few studies on the diurnal variation in cortisol levels in left-behind children and adolescents. Further research is needed to verify this speculation with more rigorous research design and sampling (e.g., including a non-left behind control group).

### Negative Family Expressiveness and HPA Axis Functioning

In the present study, we found that children living with a high NFE had a significantly lower AUC. Our results are in line with the hypocortisolism hypothesis that HPA axis activation decreases under chronic stress, resulting in lower cortisol levels (38). This is consistent with previous studies that found that adverse family factors and long-term stress could reduce cortisol levels (36, 55, 56) (Maureen et al., 2012). Specifically, for morning cortisol, previous research has found that among toddlers reared in lower income households facing higher cumulative adversity, reduced maternal warmth was correlated with blunted early morning cortisol (57). This pattern has been suggested to reflect HPA axis downregulation following excessive stress system activation, which comes with its own costs (34).

However, in contrast to our hypothesis, we found that NFE was not related to CAR or diurnal slope. A previous study also showed that the family environment, such as perceived parental rejection, was not related to CAR in healthy youth (58). The lack of association between NFE and CAR and diurnal slope in this sample of left-behind adolescents may be an indication that NFE was not sufficient to influence the physiological processes that underlie CAR and diurnal slope. It's worth noting that most of the participants in our study had both parents out. Due to the small sample size, we did not examine whether the effect of family atmosphere on children's cortisol differs across types of left-behind adolescents (e.g., both parents out vs. only one parent out). Future research should consider examine this question with a more representative sample of diverse left-behind adolescents.

### The Mediating Role of HPA Axis Functioning

Consistent with previous studies that reported a mediating role of HPA axis activity in the relation between early adversity and poor outcomes in children (45, 46), the present study found that NFE was indirectly associated with internalizing problems with diurnal cortisol secretion as a mediator in left-behind adolescents. Because of the role of the HPA axis as a core stress system that has profound effects on neurodevelopment, many studies have explored its mediating effect on adversity-psychopathology relations. For example, cortisol levels at baseline in the laboratory mediated the association between family instability (e.g., caregiver changes, variations in caregiver intimate relationships, and residential mobility) and deficits in children's effortful control at age 4 (59). However, the mediating effect of HPA axis activity has not always been found in other studies (60, 61). A number of factors may account for these inconsistent results, such as the specific types of early adversity experience and the indicators of HPA axis activity used in the studies. Even in one single study, results were mixed across different indicators of HPA axis activity. For example, in a Filipino adolescent cohort, a flatter cortisol diurnal slope, higher evening cortisol, lower total cortisol output, and lower cortisol awakening response (CAR) were associated with lower socioeconomic status across multiple developmental periods (62). To date, although converging evidence supports the mediating role of HPA axis activity in adversity–psychopathology relations, a question remains whether HPA axis activity is a key biological mechanism that is triggered by adversity and eventually affects psychopathology or whether it serves only as a marker for other biological processes that produce psychopathology (53). Additional research is needed to determine the specific meaning of different indicators of HPA axis activity and to examine the roles of other neurophysiological systems.

### Strengths and Limitations

In this short-term longitudinal study, we investigated the relations among negative family expressiveness, HPA axis functioning, and internalizing problems in left-behind adolescents. We found that NFE predicted adolescents' increased internalizing problems 6 months later after controlling for their previous internalizing problems, and that cortisol (specifically AUC) played a mediating role in this linkage. Extending the existing literature about left-behind children via a psychoneuroendocrinological perspective, the present study documents the negative consequences of NFE for at-risk youth's mental health.

Several limitations of this work should be noted. First, the sample size was small, and the study needs to be replicated with a larger number of participants before a strong conclusion can be drawn. In addition, the present sample is comprised primarily of adolescents with both parents out. Whether the findings can be generalized to other types of left-behind adolescents (e.g., only father out or only mother out) requires further research. Second, although the results in our study imply longitudinal relations from NFE to diurnal cortisol production and internalizing problems, inference of causal relations require rigorous experimental studies and ideally three waves of data are advised to establish a longitudinal mediation model. Finally, both NFE and internalizing problems were self-reported, which may induce biased views. Future research should collect data from multiple informants.

## Conclusions

In summary, negative family expressiveness is associated with a maladaptive pattern of diurnal cortisol production and internalizing problems. The relationship between NFE and internalizing problems was mediated by diurnal cortisol levels, indicating that aberrant HPA axis functioning may be an important marker for adolescents' problem behaviors. With these in mind, the development and distribution of strategies designed to attenuate feelings of NFE is of paramount importance given its concerning consequences.

## Data Availability Statement

The original contributions presented in the study are included in the article/supplementary material, further inquiries can be directed to the corresponding author/s.

## Ethics Statement

The studies involving human participants were reviewed and approved by Tianjin Normal University's Institutional Review Board. Written informed consent to participate in this study was provided by the participants' legal guardian/next of kin.

## Author Contributions

ML, LZ, and LQ designed the study. ML, QL, LQ, and FH performed the research and acquired the data. ML, FH, YY, and CZ interpreted and analyzed the data. ML, QL, YY, and LZ drafted, revised, and wrote the paper. All authors contributed to the article and approved the submitted version.

## Funding

This work was supported by the Key Project of Scientific Research Program of Tianjin Education Commission (Mental Health Education) (Grant No. 2021ZDGX11).

## Conflict of Interest

The authors declare that the research was conducted in the absence of any commercial or financial relationships that could be construed as a potential conflict of interest.

## Publisher's Note

All claims expressed in this article are solely those of the authors and do not necessarily represent those of their affiliated organizations, or those of the publisher, the editors and the reviewers. Any product that may be evaluated in this article, or claim that may be made by its manufacturer, is not guaranteed or endorsed by the publisher.

## References

[1] National Bureau of Statistics of China. National Bureau of Statistics of People's Republic of China on Major Figures of the 2010 Population Census. (2011). Available online at: http://www.chinadaily.com.cn/china/2011-04/28/content_12415526.htm (accessed June 20, 2018).

[2] Lv L Yan F Duan C Cheng M. Changing patterns and development challenges of child population in China. Populat. Res. (2018) 42:65–78.32471396

[3] Lan X Radin R. Direct and interactive effects of peer attachment and grit on mitigating problem behaviors among urban left-behind adolescents. J Child Fam Stud. (2020) 29:250–60. 10.1007/s10826-019-01580-9

[4] Hu H Lu S Huang C. The psychological and behavioral outcomes of migrant and left-behind children in China. Child Youth Serv Rev. (2014) 46:1–10. 10.1016/j.childyouth.2014.07.02134751758

[5] Sun X Tian Y Zhang Y Xie X Heath MA Zhou Z. Psychological development and educational problems of left-behind children in rural China. Sch Psychol Int. (2015) 36:227–52. 10.1177/014303431456666930615250

[6] Wu J Zhang J. The effect of parental absence on child development in rural China. Asian Econ Policy Rev. (2017) 12:117–34. 10.1111/aepr.12166

[7] Zhao J Liu X Wang M. Parent-child cohesion, friend companionship and left-behind children's emotional adaptation in rural China. Child Abuse Negl. (2015) 48:190–9. 10.1016/j.chiabu.2015.07.005 26190190

[8] Zhao F Yu G. Parental migration and rural left-behind children's mental health in China: a meta-analysis based on mental health test. J Child Fam Stud. (2016) 25:3462–72. 10.1007/s10826-016-0517-3

[9] Tang W Wang G Hu T Dai Q Xu J Yang Y. Mental health and psychosocial problems among Chinese left-behind children: a cross-sectional comparative study. J Affect Disord. (2018) 241:133–41. 10.1016/j.jad.2018.08.017 30121025

[10] Cicchetti D Sroufe LA. The past as prologue to the future: the times, they've been a-changin'. Dev Psychopathol. (2000) 12:255–64. 10.1017/S0954579400003011 11014738

[11] Fleming CB Mason WA Mazza JJ Abbott RD Catalano RF. Latent growth modeling of the relationship between depressive symptoms and substance use during adolescence. Psychol Addict Behav. (2008) 22:186–97. 10.1037/0893-164X.22.2.186 18540716

[12] Mesman J Bongers IL. Preschool developmental pathways to preadolescent internalizing and externalizing problems. J Child Psychol Psychiatry. (2001) 42:679–89. 10.1111/1469-7610.00763 11464972

[13] Lewinsohn PM Rohde P Seelry JR. Major depressive disorder in older adolescents: prevalence, risk factors, and clinical implications. Clin Psychol Rev. (1998) 18:765–94. 10.1016/S0272-7358(98)00010-5 9827321

[14] Bronfenbrenner U. The Ecology of Human Development: Experiments by Nature and Design. Cambridge, MA: Harvard University Press (1979).

[15] Bronfenbrenner U Ceci SJ. Nature-nurture reconceptualized in developmental perspective: a bioecological model. Psychol Rev. (1994) 101:568–86. 10.1037/0033-295X.101.4.568 7984707

[16] Milojevich HM Haskett ME. Longitudinal associations between physically abusive parents' emotional expressiveness and children's self-regulation. Child Abuse Negl. (2018) 77:144–54. 10.1016/j.chiabu.2018.01.011 29353718 PMC5857221

[17] Dunsmore JC Halberstadt AG. How does family emotional expressiveness affect children's schemas? New Direct Child Dev. (1997) 77:45–68. 10.1002/cd.23219977704 9457805

[18] Eisenberg N Gershoff ET Fabes RA Shepard SA Cumberland AJ Losoya SH. Mothers' emotional expressivity and children's behavior problems and social competence: mediation through children's regulation. Dev Psychol. (2001) 37:475–90. 10.1037/0012-1649.37.4.475 11444484

[19] Chen SH Zhou Q Eisenberg N Valiente C Wang Y. Parental expressivity and parenting styles in chinese families: prospective and unique relations to children's psychological adjustment. Parent Sci Pract. (2011) 11:288–307. 10.1080/15295192.2011.613725 23226715 PMC3513915

[20] Lu Y. Education of children left behind in rural China. J Marriage Fam. (2012) 74:328–41. 10.1111/j.1741-3737.2011.00951.x 24163479 PMC3806142

[21] Wen M Lin D. Child development in rural china: children left behind by their migrant parents and children of nonmigrant families. Child Dev. (2012) 83:120–36. 10.1111/j.1467-8624.2011.01698.x 22181046

[22] Ling H Fu E Zhang J. Effects of separation age and separation duration among left-behind children in China. Soc Behav Pers. (2015) 43:241–54. 10.2224/sbp.2015.43.2.24133059932

[23] Doom JR Hostinar CE VanZomeren-Dohm AA Gunnar MR. The roles of puberty and age in explaining the diminished effectiveness of parental buffering of HPA reactivity and recovery in adolescence. Psychoneuroendocrinology. (2015) 59:102–11. 10.1016/j.psyneuen.2015.04.024 26047719 PMC4490054

[24] Doom JR Doyle CM Gunnar MR. Social stress buffering by friends in childhood and adolescence: effects on HPA and oxytocin activity. Soc Neurosci. (2017) 12:8–21. 10.1080/17470919.2016.1149095 26899419 PMC5538015

[25] Engel ML Gunnar MR. The development of stress reactivity and regulation during human development. Int Rev Neurobiol. (2020) 150:41–76. 10.1016/bs.irn.2019.11.003 32204834

[26] Lupien SJ McEwen BS Gunnar MR Heim C. Effects of stress throughout the lifespan on the brain, behaviour and cognition. Nat Rev Neurosci. (2009) 10:434–45. 10.1038/nrn2639 19401723

[27] Clow A Hucklebridge F Stalder T Evans P Thorn L. The cortisol awakening response: more than a measure of HPA axis function. Neurosci Biobehav Rev. (2010) 35:97–103. 10.1016/j.neubiorev.2009.12.011 20026350

[28] Vargas I Lopez-Duran N. Dissecting the impact of sleep and stress on the cortisol awakening response in young adults. Psychoneuroendocrinology. (2014) 40:10–6. 10.1016/j.psyneuen.2013.10.009 24485471

[29] Kalsbeek A van der Spek R Lei J Endert E Buijs RM Fliers E. Circadian rhythms in the hypothalamo-pituitary-adrenal (HPA) axis. Mol Cell Endocrinol. (2012) 349:20–9. 10.1016/j.mce.2011.06.042 21782883

[30] Kuhlman KR Repetti RL Repetti RL Reynolds BM Robles TF. Change in parent-child conflict and the HPA-axis: where should we be looking and for how long? Psychoneuroendocrinology. (2016) 68:74–81. 10.1016/j.psyneuen.2016.02.029 26963373 PMC5403246

[31] McEwen BS. Effects of adverse experiences for brain structure and function. Biol Psychiatry. (2000) 48:721–31. 10.1016/S0006-3223(00)00964-1 11063969

[32] Lupien SJ King S Meaney MJ McEwen BS. Can poverty get under your skin? Basal cortisol levels and cognitive function in children from low and high socioeconomic status. Dev Psychopathol. (2001) 13:653–76. 10.1017/S0954579401003133 11523853

[33] Evans GW Kim P. Childhood poverty and health-cumulative risk exposure and stress dysregulation. Psychol Sci. (2007) 18:953–7. 10.1111/j.1467-9280.2007.02008.x 17958708

[34] Fries E Hesse J Hellhammer J Hellhammer DH. A new view on hypocortisolism. Psychoneuroendocrinology. (2005) 30:1010–6. 10.1016/j.psyneuen.2005.04.006 15950390

[35] Bruce J Fisher PA Pears KC Levine S. Morning cortisol levels in preschool-aged foster children: differential effects of maltreatment type. Dev Psychobiol. (2009) 51:14–23. 10.1002/dev.20333 18720365 PMC2644049

[36] Tian T Young CB Zhu Y Xu J He Y Chen M. Socioeconomic disparities affect children's amygdala-prefrontal circuitry via stress hormone response. Biol Psychiatry. (2021) 90:173–81. 10.1016/j.biopsych.2021.02.002 33832707

[37] Holsboer F. Stress, hypercortisolism and corticosteroid receptors in depression: implicatons for therapy. J Affect Disord. (2001) 62:77–91. 10.1016/S0165-0327(00)00352-9 11172875

[38] Gunnar MR Vazquez DM. Low cortisol and a flattening of expected daytime rhythm: potential indices of risk in human development. Dev Psychopathol. (2001) 13:515–38. 10.1017/S0954579401003066 11523846

[39] Lippold MA McHale SM McHale SM Davis KD Almeida DM King RB. Experiences with parents and youth physical health symptoms and cortisol: a daily diary investigation. J Res Adolesc. (2016) 26:226–40. 10.1111/jora.12186 27231418 PMC4876874

[40] Nederhof E Marceau K Shirtcliff EA Hastings PD Oldehinkel AJ. Autonomic and adrenocortical interactions predict mental health in late adolescence: the TRAILS study. J Abnorm Child Psychol. (2015) 43:847–61. 10.1007/s10802-014-9958-6 25421943

[41] Saridjan NS Velders FP Jaddoe VW Hofman A Verhulst FC Tiemeier H. The longitudinal association of the diurnal cortisol rhythm with internalizing and externalizing problems in pre-schoolers. Gen R Study Psychoneuroendocrinol. (2014) 50:118–29. 10.1016/j.psyneuen.2014.08.008 25202831

[42] Lopez-Duran NL Kovacs M George CJ. Hypothalamic-pituitary-adrenal axis dysregulation in depressed children and adolescents: a meta-analysis. Psychoneuroendocrinology. (2009) 34:1272–83. 10.1016/j.psyneuen.2009.03.016 19406581 PMC2796553

[43] Shirtcliff EA Essex MJ. Concurrent and longitudinal associations of basal and diurnal cortisol with mental health symptoms in early adolescence. Dev Psychobiol. (2008) 50:690–703. 10.1002/dev.20336 18726897 PMC2660275

[44] Chen C Nakagawa S An Y Ito K Kitaichi Y Kusumi I. The exercise-glucocorticoid paradox: How exercise is beneficial to cognition, mood, and the brain while increasing glucocorticoid levels. Front Neuroendocrinol. (2016) 44:83–102. 10.1016/j.yfrne.2016.12.001 27956050

[45] van Goozen SHM Fairchild G Snoek H Harold GT. The evidence for a neurobiological model of childhood antisocial behavior. Psychol Bull. (2007) 133:149–82. 10.1037/0033-2909.133.1.149 17201574

[46] Martin CG Kim HK Bruce J Fisher PA. Child diurnal cortisol rhythms, parenting quality, and externalizing behaviors in preadolescence. Psychoneuroendocrinology. (2014) 40:170–80. 10.1016/j.psyneuen.2013.11.015 24485489 PMC3935801

[47] Marceau K Laurent HK Neiderhiser JM Reiss D Shaw DS Natsuaki MN. Combined influences of genes, prenatal environment, cortisol, and parenting on the development of children's internalizing versus externalizing problems. Behav Genet. (2015) 45:268–82. 10.1007/s10519-014-9689-z 25355319 PMC4416104

[48] Halberstadt AG. Family socialization of emotional expression and nonverbal-communication styles and skills. J Pers Soc Psychol. (1986) 51:827–36. 10.1037/0022-3514.51.4.827

[49] Liu A Wang M Zhang J Xing X. Relationship between family expressiveness and preschool children‘s anxiety. Chin J Clin Psychol. (2009) 17:465–7. 10.16128/j.cnki.1005-3611.2009.04.010

[50] Achenbach TM Rescorla LA. Manual for the ASEBA School-Age Forms & Profiles. Burlington, VT: University of Vermont, Research Center for Children, Youth, & Families (2001).

[51] Joshua MS Margit CO Amy AG Delwyn C Laura SP Clemes K. Individual differences in the diurnal cycle of cortisol. Psychoneuroendocrinology. (1997) 22:89–105. 10.1016/S0306-4530(96)00039-X 9149331

[52] Pruessner JC Kirschbaum C Meinlschmid G Hellhammer DH. Two formulas for computation of the area under the curve represent measures of total hormone concentration versus time-dependent change. Psychoneuroendocrinology. (2003) 28:916–31. 10.1016/S0306-4530(02)00108-7 12892658

[53] Koss KJ Gunnar MR. Annual research review: early adversity, the hypothalamic-pituitary-adrenocortical axis, and child psychopathology. J Child Psychol Psychiatry. (2018) 59:327–46. 10.1111/jcpp.12784 28714126 PMC5771995

[54] Zeiders KH Doane LD Adam EK. Reciprocal relations between objectively measured sleep patterns and diurnal cortisol rhythms in late adolescence. J Adolesc Health. (2011) 48:566–71. 10.1016/j.jadohealth.2010.08.012 21575815 PMC3179910

[55] Fisher PA Van Ryzin MJ Gunnar MR. Mitigating HPA axis dysregulation associated with placement changes in foster care. Psychoneuroendocrinology. (2011) 36:531–9. 10.1016/j.psyneuen.2010.08.007 20888698 PMC3610565

[56] Raffington L Schmiedek F Heim C Shing YL. Cognitive control moderates parenting stress effects on children's diurnal cortisol. PLoS ONE. (2018) 13:e0191215. 10.1371/journal.pone.0191215 29329340 PMC5766146

[57] Zalewski M Lengua LJ Kiff CJ Fisher PA. Understanding the relation of low income to HPA-axis functioning in preschool children: cumulative family risk and parenting as pathways to disruptions in cortisol. Child Psychiatry Hum Dev. (2012) 43:924–42. 10.1007/s10578-012-0304-3 22528032 PMC3621874

[58] Marsman R Nederhof E Rosmalen J Oldehinkel AJ Ormel J Buitelaar J. Family environment is associated with HPA-axis activity in adolescents. The trails study. Biol Psychol. (2012) 89:460–6. 10.1016/j.biopsycho.2011.12.013 22212280

[59] Sturge-Apple ML Davies PT Cicchetti D Hentges RF Coe JL. Family instability and children's effortful control in the context of poverty: Sometimes a bird in the hand is worth two in the bush. Dev Psychopathol. (2017) 29:685–96. 10.1017/S0954579416000407 27580955

[60] Demireva P Suhr J Heffner K. Cortisol is inversely related to primacy, but not recency effect in list learning and recall. Clin Neuropaychol. (2008) 22:397.

[61] Marsman R Rosmalen JGM Oldehinkel AJ Ormel J Buitelaar JK. Does HPA-axis activity mediate the relationship between obstetric complications and externalizing behavior problems? The TRAILS study. Eur Child Adolesc Psychiatry. (2009) 18:565–73. 10.1007/s00787-009-0014-y 19353232 PMC2721131

[62] Desantis AS Kuzawa CW Adam EK. Developmental origins of flatter cortisol rhythms: socioeconomic status and adult cortisol activity. Am J Hum Biol. (2015) 27:458–67. 10.1002/ajhb.22668 25753264

